# Designing a Low-Fat Food Packaging: Comparing Consumers’ Responses in Virtual and Physical Shopping Environments

**DOI:** 10.3390/foods10020211

**Published:** 2021-01-21

**Authors:** Natalia Vila-López, Ines Kuster-Boluda, Adrian Alacreu-Crespo

**Affiliations:** 1 Departamental Facultad de Economia (1er piso), Department of Comercialización e Investigación de Mercados. Edif, Avda Tarongers, s/n, Universidad de Valencia, 46022 Valencia, Spain; Ines.Kuster@uv.es; 2 Department of Emergency Psychiatry and Post-Acute Care, Academic Hospital Montpellier, 43090 Montpellier, France; adrian.alacreu@uv.es

**Keywords:** emotions, cognition, physical, virtual, packaging

## Abstract

This paper aims to test to what extent emotional responses towards a low-fat product presented virtually converge with emotional responses toward this product when presented physically. Second, we want to probe if low-order emotions (physiological/unconscious responses) and high-order emotions (cognitive/conscious responses) converge to explain healthy product choices. To this end, 83 young participants were engaged in our experiment. Two packaging design variables were manipulated with the help of a real company (the color and the message), so that six different packages were created. Two different buying contexts were simulated: A virtual context and a physical context. Physiological responses were continuously recorded in both contexts (heart rates, electro-dermal responses, and eye muscle reactions). At the end, participants provided cognitive responses in a questionnaire concerning the selected package. Our results have demonstrated that low-order emotions remain stable (from a virtual to a physical environments). Virtual simulations elections and real product elections are correlated (X^2^ = 40.493; *p* < 0.02). Physiological and cognitive responses do not converge. Correlations between unconscious responses (low-order emotions) and self-reported measures (high-order emotions) was contrary to expectations (negative sign). Only low-order emotions explain product choices. On the contrary, real packaging choice and high-order emotions correlated inversely (the t values were significant but negative).

## 1. Introduction

The role of emotions in consumer behavior models has been increasing during the last decades [1]. In this field of research, this paper tries to progress in two lines of research.

On one side, the Theory of Consumer Learning [2] supports the idea that consumers’ emotions appear at very early stages of the consumption process, not just when the real product is shown at the point of sale. This means that when consumers search for product information, they start to learn about it, and, consequently, to feel something for the product. What they start to feel towards a product early presentation on a computer screen will reinforce what they finally feel towards the real product. That is, what users have learnt previously about a product on a computer (“zero moment of truth: ZMOT”) will affect the real emotions they feel towards the physical/real product. Thus the previous emotions experienced towards a new product presented virtually and the final emotions towards the same product presented physically in a shop are expected to converge.

On another side, the Affective-Cognitive Model of consumer decision making [3], supports the idea that low-order emotions (physiological responses) and high-order emotions (cognitive/verbal responses) work together. This is because “on exposure to alternatives in a choice task, two types of processes may be engendered, one affective in nature and the other cognitive in nature. This means that emotions could be separated into two main groups: Low-order emotions (unconscious) and higher-order emotions (conscious). Lower-order affective reactions arise from relatively automatic processes while higher-order affective reactions arise from relatively more controlled, higher-order processes involved in thinking, reasoning, and consciousness” (p. 279). Both groups of emotions, lower-order and higher-order, can be useful to explain product choices in a spontaneous and controlled way, respectively.

Taking both lines of research as a starting point, the main objectives of this paper are twofold. First, we wish to demonstrate the convergence between emotional responses towards fictitious stimuli (representing a healthy product) shown on a computer screen and emotional responses towards the real products shown in a physical environment. The final purpose is to demonstrate in the low-fat food industry to what extent virtual packaging choices could anticipate real packaging choices. To this end, consumers’ low-order emotions were recorded in two different time moments that represent two different shopping environments (virtual and physical). Second, we analyzed the relationships between low-order (uncontrolled) emotions and higher-order (controlled) responses in a questionnaire to better understand the link between physiological and cognitive responses to explain healthy-product choices.

It has been suggested that consumers’ responses to product trials may be influenced by prior ad (or other information) exposure [4]. As [5] remark, “despite the a-knowledge role of physiological, unconscious arousal in shaping consumers’ evaluations and behaviors, most of the research on this issue has been conducted in psychology and neuroscience and has not been widely acknowledged in marketing yet”. As [4]) report, relatively few studies have investigated how previous information and virtual product trials are (and can be) used by consumers to anticipate their final elections in physical shopping environments. On another side, Shiv et al. [3] there is still limited research on the convergence “between lower-order affective reactions, arising from relatively automatic processes, and higher-order affective reactions, arising from relatively more controlled, higher-order processes involved in thinking, reasoning, and consciousness”. As they underline, “a unified conception of the interface between affect, cognition and behavior does not exist yet” (p. 252). As [6] remark, “individuals’ perceptions of their environment are frequently regulated by an unconscious mechanism that does not necessarily involve rational thinking”.

To cover both literature gaps, this paper extends existing research with two main contributions. On one side, we explain consumers’ choices through a longitudinal approach that compares low-order emotions (heart rate, skin conductance, and eye movements) felt in two different time moments: First, when several virtual stimuli (representing alternative low-fat product packages) are early shown using screen presentations; and, second, when real product packages are displayed. The use of these tools represent a promising field of research [7]. On another side, we have added in the study the measurement of high-order emotions (cognitive responses in a questionnaire) to compare to what extent low- and high-order emotions lead to similar results in terms of healthy product trials. 

### 1.1. Convergence of Low-Order Emotions in Two Different Contexts: Virtual and Physical Choosing Environments

“Measuring food-evoked emotions can improve the understanding of food choice, consumption, and consumer behavior” [1]. As these authors indicate, emotions are not single responses but series of dynamic events that unfold over time. In this paper, two different moments will be considered in the consumption process in order to explain final choices: (i) Emotional responses felt towards virtual stimuli presentations representing fictitiously different product packages (moment one); and (ii) emotional responses felt towards real product package exhibitions (moment two). In both scenes, consumers were forced to choose the option that they would really buy to feel healthy.

Our starting point is based on the work of [2]), supporting the convergence between consumers’ responses in virtual and physical environments. As these authors report, the Internet serves as a more powerful medium than traditional print or broadcast media because consumers are able to interact with products, thus simulating a new form of experience—a virtual experience. However, very little research has explored the impact of virtual experience, especially in combination with direct/physical (product trial) experience. “A virtual experience is a vivid, involving, active, and affective psychological state that consumers encounter when interacting with 3D products in a computer-mediated environment” (p. 569). As they conclude, “the virtual experience from 3D product visualization is more similar to direct experience” than other forms of communication (p. 568). From this approach, we can conclude that stimuli pre-trial (shown on a computer screen) influences physical products post-trial. That is, a significant stimulus-product trial interaction occurs. In the words of [4], “after an ad exposure, consumers form tentative beliefs based on the attribute information in the ad that then serve as hypotheses about the brand to be tested in a subsequent product trial”. From this approach, real product trial behaviors are the result of more careful and focused processing of the pre-trial information, which was obtained watching an ad or a computer screen. So, pre-trial selection of a virtual stimulus on a computer screen will directly influence the physical product trial.

From a different approach, [3]) support the opposite point of view, suggesting that virtual and physical responses will not converge. As these authors report, presenting respondents with photographs of the alternatives (i.e., virtual presentations), “rather than the real alternatives, is likely to reduce the vividness of the options and hence the intensity of positive affect experienced by respondents”. From this approach, low-order emotions in virtual-trial contexts will be lower than low-order emotions felt in physical trial experiences.

In the present work, we have followed the first approach, supporting the line of research started by the Theory of Consumer Learning, which maintains that there will be convergence between virtual choices and physical choices [2]. From this approach, Ref. [1] underline the fact that emotions tend to be stable. That is, as time goes on, what people feel at the beginning (moment one) becomes reinforced with time (moment two). From this perspective, we anticipate that low-order emotions will remain stable. That is, virtual choices (moment one) and real product choices (moment two) will be accompanied by the same type of emotions. Thus:

**Hypothesis** **1** **(H1).** *Low-order emotions (physiological responses) will remain stable, meaning that emotional responses towards a healthy package shown on a virtual simulation (moment one) converge with emotional responses toward this product when presented physically (moment 2).*

### 1.2. Convergence between Low-Order Emotions and High-Order Emotions to Explain Product Choices

The link between emotions and consumption behavior has been deeply studied [8,9]. The seminal work of [10] Shiffrin and Schneider (1977) differentiates between controlled and automatic human information processing. From this first approach, it is reinforced that “after a long period that was dominated by cognitive theories of economic behavior, we are now witnessing a period in which affect gets a prominent place in our theories” (p. 252). From this line of research, its supported that affect (low-order emotions) will determine cognition (high-order emotions), so that product trials could be explained using both kinds of responses interchangeably. 

In contrast, the revision of [3] suggests that low-order emotions (affect) and high-order emotions (cognition) do not always work together. As these authors explain, if the availability of processing resources is constrained (i.e., no information about the product has been provided), the consumer’s behavior is likely to be influenced primarily by the affective reactions elicited by the task (low-order emotions). He/she decides emotionally. Conversely, if processing resources are available (i.e., prospects, advertisements, or seller explanations), a healthy consumer’s behavior is likely to be influenced primarily by cognition arising from higher-order processes (high-order emotions). He/she decides cognitively. In the same vein, [5] have also found, using a sample of 160 participants, that conscious and unconscious arousal are two independent emotional responses, and they influence attitude toward the product differently, which means that physiological measures and verbal measures can lead to different results. A comparison of these two different measurement tools (conscious and unconscious) was also study by [11], obtaining also different results. Following this proposal, we would like to demonstrate that:

**Hypothesis** **2** **(H2).** *Low-order emotions (physical responses) and high-order emotions (cognitive responses) will not converge to explain healthy product choices.*

## 2. Materials and Methods

### 2.1. Sample

This study engaged 83 healthy, normal-weight participants (46 men, 37 women; mean age 21.5 years, standard deviation 2.44). All participants consumed the healthy product categories presented in this study and reported no illness. This sample size is adequate for the purpose of the study [12]. This young group of the population was chosen because there has been little academic research focused on what types of digital marketing strategies are preferred by this segment and which ones influence its behavior [13]. Participants were contacted personally, in a public street, by a representative from an external company at the door of five different facilities and a public hospital. Once they had confirmed that they were used to buying the products considered in our study, they were invited to take part in our experiment and, if they accepted, they were taken to the laboratory on the campus of a Spanish university. The experienced the product virtual and physically in two different environmental situations. “In Spain, the study developed by [14] shows that people sharing virtual and physical distribution channels before buying represent 52% of the population”. 

### 2.2. Product 

**Figure 1:**
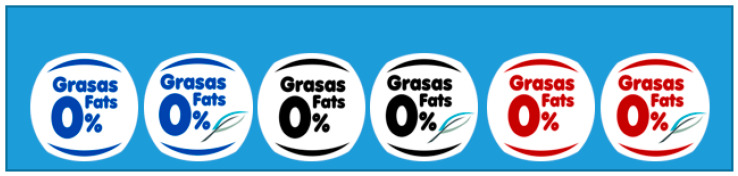
Six prototypes combining 3 colors and 2 messages.

With the help of real company, we manipulated two packaging variables for our experiment: The color and the message. Thus, six packaging designs were created following a fractionated factorial experimental design. We used a real brand name, but applied two a new healthy food product that wanted to be tested. The company prepared the real packages that needed to be tested with the help of a graphic designer. The description of the product was invariant. Figure 1 shows a simplification of the six different packages that were created.

### 2.3. Experimental Design 

Two different environments were analyzed: A virtual scene using screen presentations on a computer and a physical scene using real products. In both environments, two packaging variables (color and message on the label) were manipulated. As in the work of [2], in our experiment consumers’ virtual experience shared the properties of direct experience because they could interact with products through multisensory iconic representations that simulated the properties of direct experience. As these authors state, a virtual experience is a simulation of a real or physical experience, which occurs within a computer-mediated environment.

#### 2.3.1. Experiment 1 (Moment/Phase 1): Virtual Stimuli Presentations (Virtual Environment)

Following [15] we used an experimental design in which two packaging attributes were manipulated orthogonally: A visual cue (the color of the package) and a graphic cue (a simple versus a complex claim; the second reinforced product healthiness by adding the image of a leaf to the simple claim). 

The design of the experiment was a 3 (colors) × 2 (messages) within-subjects design, which resulted in six virtual packaging screen presentations (Figure 1). In line with [16], Hurley, Hutcherson, Tonkin, Dailey, and Rice (2015), we tried to eliminate the potential order effects that could result from the way in which the packaged products were presented. Five treatment blocks with different sequences of screen presentations were therefore prepared, and each participant was exposed to each of the six product packaging simulations five times. Specifically, three phases were followed to collect the data:

1. Participants were recruited following verbal checks that they were users of the products that would later be presented. When arriving at the laboratory, the participants were asked to complete a survey in order to eliminate inappropriate participants (those who smoked more than 10 cigarettes per day, those who took illegal drugs or who were on medication, and those who had any kind of heart or psychiatric illness).
2. The experiment was explained to those who passed the previous phase, and they signed an informed consent form that had been approved by the Research Ethics Committee of the university. The participants were then connected to the physiological sensors. To minimize interference, the participants were instructed to avoid moving the electrodes.
3. The task to be performed by the subjects was initiated. This consisted of viewing on a computer a series of virtual product package presentations that were differentiated by their color and message characteristics. Each participant was shown a pool of six different virtual stimuli five times. To this end, a total of five repetitions for each virtual stimulus was done in a random order, making a total of 30 screen presentations per person. Each screen was shown for 11 s, giving a total of 330 s (5.5 min) of exposure. After the 30 screen presentations, one last presentation showed the six virtual designs in a random order, forcing the subject to select the one for which he/she felt the greatest preference.

During this experiment, three physiological variables were continuously recorded (heart rates, electro-dermal responses, and eye muscle reactions). Thus, consumers’ responses towards each virtual package were computed for each person five times.

#### 2.3.2. Experiment 2 (Moment/Phase 2): Real Product Presentations (Physical Environment)

Upon the completion of phase 1, experiment 2 followed the same procedure of experiment 1 but used six real packaging concepts instead of six virtual screen presentations. Once again, in this second experiment, we continuously recorded heart rates, skin responses, and eye muscle responses when real products were presented, changing the order of presentation. Thus, consumers’ responses towards each real package were computed for each person five times.

Then, electrodes were removed and the participants finished providing cognitive responses about a selected packaging, evaluating the packaging through questions on a questionnaire. As in diverse studies [16,17], the physiological assessment was carried out first, and then the self-report measures (cognitive response) were administered, because physiological reactions are spontaneous and decay rather fast.

All the sessions, including both experiments, lasted for one hour for each person.

### 2.4. Data Collection Methods and Data Reduction

Based on [2] two kinds of responses were collected: Low-order emotions (physiological responses based on heart, skin, and eye muscle reactions) and high-order emotions (self-report measures on a questionnaire). 

To measure low-order emotions (physiological responses), three kind of responses were used: Heart rate (heart response), skin conductance (dermal response), and corrugator electromyogram (eye muscle response). The latter was added because “facial electromyography (EMG) is a more precise measure of facial expressions” [18]. For heart rates (HR: Heart rates), scores were computed by determining, for each participant and each stimulus, the maximum deceleration from baseline in the first 3-s of picture viewing and the maximum acceleration from baseline in the last 3-s of stimulus viewing [19]. Given that each stimulus was presented five times (altering the order of presentation in the pool of competing stimuli), an average was calculated for the maximums and the minimums. We repeated this procedure with heart responses collected in our second experiment, in this case using physical products instead of virtual screen presentations.

1. For skin conductance (EDA: Electro dermal activity), the maximum change occurring between 0 and 4-s after stimulus onset was scored. Also, the minimum value between 4- and 6-s after stimulus onset was scored [19]. Both variables were obtained for each person and each virtual stimulus. Given that each stimulus was presented five different times (altering the order of presentation in the pool of competing stimuli), an average was calculated for the maximums and the minimums. We repeated this procedure with skin responses collected in our second experiment, in this case using physical products instead of virtual screen presentations.
2. For eye muscle responses (Facial electromyography, EMG: Reactions in corrugator and zygomatic eye muscles), the average change over the 6-s picture period was used to estimate reactivity [19]. Given that each stimulus was presented five times, an average of the medians was calculated. We repeated this procedure with eye responses collected in our second experiment, in this case towards real products.

To collect these tree kind of physiological responses, PowerLab/16SP equipment (ADInstruments) was used. Based on previous studies, electrodes were placed on the finger (to record heart rate), upon the sweat-sensible places of the palm of the hand (to record skin conductance), and on two eye muscles (to record facial responses).

To collect cognitive responses, the participants were encouraged to evaluate the chosen option using the SAM scale (Self-Assessment Manikin) in a questionnaire. This is a nonverbal pictorial scale that measures the pleasure, activation, and dominance associated with a subject’s affective reaction to a wide variety of stimuli or products [20]. More specifically, nine items were used to evaluate the selected packaging: Three items measured valence (pleasure), three items measured activation (arousal), and three items measured domination. Regarding valence, activation, and domination, we proceeded by averaging the responses over the different stimuli [21].

## 3. Results and Discussion

### 3.1. Convergence of Low-Order Emotions in Two Different Contexts: Virtual and Physical Choosing Environments

The main diagonal of Table 1 shows the convergence between the trial of virtual stimuli and real products. The highest convergence occurs for the blue packaging. In this case, 66.67% of the consumers who chose the blue packaging with a simple label in the virtual space also chose the blue packaging with a simple label in the physical context. The second and third highest convergence between virtual trial and real trial are for the red packaging with a reinforced label (adding a leaf to the message) (62.50%) and for the blue packaging with a leaf (47.92%).

Thus, regarding product selection, we conclude virtual stimuli choices (moment 1) and real packaging choices (moment 2) do not totally converge. However, a positive and significant relationship exists between both contexts: Virtual simulations elections and real product elections (X^2^ = 40.493; *p* < 0.02). Therefore, previous elections in a virtual space could be useful to anticipate posterior elections in a physical context in the significant way shown by Table 1.

Investigating these choices in more detail, our results show that:

1. In the virtual context (experiment 1 in moment/phase 1), the preferred option for a low-fat product was a blue packaging with an elaborated label (leaf) (61.4% of the sample), followed by the black packaging with an elaborated label (13.3%) and the red packaging with elaborated label (10.8%). Thus, in the virtual context, the elaborated label—a light leaf to reinforce the low-fat message—was important to stimulate the election a low-fat product. In this scene, the content (message in the label) was more important than the aesthetics (color). The most preferred options shared a common message (light leaf), but not a common color (Table 1 and Table 2).
2. In the physical context (experiment 2 in moment/phase 2), consumers again preferred the blue packaging with an elaborated label (38.96%), followed by the blue packaging with a simple message (22.08%). Thus, in this context, the blue color proved to be efficacious. Thus, the form/aesthetics was more important than the message when healthy products were shown physically. The most preferred options shared a common color (blue), but not a common message (Table 1).

Focusing now on low-order emotions (unconscious responses), three indicators were used in both environments: Heart rate, electro-dermal activity, and electromyography (eye muscle reactions). For each person, these three variables were recorded five times per packaging concept (altering the order of the presentation). Then, an average indicator was obtained for each packaging concept and each person. This procedure was followed twice, in the virtual environment (experiment 1 in moment/phase 1) and the physical environment (experiment 2 in moment/phase 2):

Results (Table 2) show that:

1. Regarding heart rate indicators (heart responses), the correlation between the blue virtual stimulus and the blue real packaging was high and significant. This also occurred for all correlations between the rest of the virtual stimuli and real packages.
2. Regarding electro-dermal activity (skin responses), the correlation between the blue virtual stimulus and the blue real packaging was high and significant. This also occurred for the rest of the packages.
3. Regarding electromyography indicators (eye reactions), the correlation between the blue virtual stimulus and the blue real packaging was high and significant. This also occurred for all the packaging options except for the blue packaging with the leaf.

In sum, the correlations between the three variables that were recoded to measure low-order emotions towards virtual stimulus and towards real products were positive and significant. Thus we can accept H1, stating that low-order emotions will remain stable, which means that emotional responses towards a low-fat product presented simulated virtually (moment/phase 1) converge with emotional responses toward the real product when created and presented physically (moment/phase 2)

### 3.2. Convergence between Low-Order Emotions (Physiological Responses) and High-Order Emotions (Cognitive Responses) to Explain Product Choices

To measure high-order emotions, a questionnaire was used to evaluated three factors: Valence, activation, and dominance. A total of nine items were used to evaluate the packaging: Three items measured valence, three items measured activation, and three items measured domination. Table 3 shows the average valuation of each packaging concept in each item. Three main points can be concluded:

1. Regarding valence (3 items), the mean values of the three items differed significantly among the six packages. A negative relationship was identified, meaning that high means of valence items corresponded to less chosen packages. That is, the higher the positive valence, the lower the ranked packaging choice.
2. Regarding activation (3 items), the mean values of just one item differed significantly among the six packages. A negative relationship was identified for this item, meaning that high means of activation corresponded to less chosen packages. For the other two items measuring activation, package differences were not identified.

**Table 3:** High-order emotions to explain product trial: Mean differences among real products.

Product Chosen		Higher Order Emotions: Valence, Activation, and Domination					
**1**		**Valence 1** **Mean**	***t***	**Activation 1** **Mean**	***t***	**Domination 1** **Mean**	***t***
38.96%	BluePlume	1.9833	−8.663 **	2.9655	−3.891 **	3.1765	−1.604
22.08%	Blue	1.8529		2.5588		3.1207	
19.48%	RedPlume	2.2333		2.4667		2.9000	
9.09%	BlackPlume	1.8571		3.0000		4.1429	
7.79%	Red	1.3000		2.5833		3.3000	
2.60%	Black	1.2500		2.2500		4.0000	
**2**		**Valence 2** **mean**	***t***	**Activation 2** **Mean**	***t***	**Domination 2** **Mean**	
38.96%	BluePlume	2.5667	−5.798 **	3.8000	0.489	2.8667	−3.314 **
22.08%	Blue	2.2059		3.3235		2.8824	
19.48%	RedPlume	2.5333		3.5000		3.0333	
9.09%	BlackPlume	3.0000		3.6429		2.7143	
7.79%	Red	1.9000		3.9167		2.4000	
2.60%	Black	1.5000		3.7500		3.7500	
**3**		**Valence 3** **mean**	***t***	**Activation 3** **Mean**	***t***	**Domination 3** **Mean**	
38.96%	BluePlume	2.4167	−6.197 **	3.4333	−0.327	3.0167	−2.563 *
22.08%	Blue	2.2059		3.4412		3.0588	
19.48%	RedPlume	2.4333		3.4667		3.1333	
9.09%	BlackPlume	2.4286		3.1429		2.7143	
7.79%	Red	1.9167		3.8333		2.5000	
2.60%	Black	1.7500		4.0000		4.0000	

** *p* < 0.01; * *p* < 0.05.

Regarding domination (3 items), the mean values of the three items differed significantly among the six packages. A negative relationship was identified for the items; thus the same inverse association was obtained with domination and product choice.

In sum, the t values were significant and negative for seven items, which means that “real packaging choice” and “high-order emotions” correlated inversely. For packaging whose choice preference was greater, the evaluation on the questionnaire was poor (low valence, activation, and domination). More specifically, the highest values of valence, activation, and domination in most of the cases were for the less chosen packages. Thus, cognition (high-order emotions) was not helpful in explaining low-fat product choices, because obtaining higher values on the questionnaire (for valence, activation, or domination) did not equate to greater product choice.

Finally, and following previous literature [9,22], correlations between unconscious responses (low-order emotions) and self-reported measures (high-order emotions) were calculated to better understand product choices. We can appreciate that the highest number of significant correlations between high-order emotions and low-order emotions occurred for the most chosen packaging: Blue color with an elaborated label (leaf to reinforce the low-fat claim). However, the sign of the correlations was contrary to expectations. More specifically, for the blue packaging with an elaborated message:

1. Heart rate (low-order emotion) and the items’ evaluation on a questionnaire (high-order emotion) were inversely correlated (Table 4). Positive correlations were expected based on previous literature [23,24] but negative values were obtained. This means that heart rate was augmented while the opinions on the questionnaire diminished. That is, if heart rate acceleration is associated with the extent to which individuals increase attention to potentially interesting stimuli [23], this should correspond to better opinions on a questionnaire (conscious emotions). For the most chosen packaging in our study (blue packaging with elaborated label), heart rate did not decay because it was the preferred option, but curiously the evaluations in the questionnaire for this packaging were not outstanding.
2. Skin responses (low-order emotion) and the items’ evaluation on the questionnaire (high-order emotion) were positively correlated (Table 4), while negative correlations were expected based on previous literature [23]. In our results, we can see that packaging with lower skin responses (unconscious emotions) corresponded to lower cognitive evaluations (conscious emotions). Consequently, lower dermal responses should correspond to most chosen packages, which should therefore match with high ratings on a questionnaire for this option. This did not occur in our experiment. The preferred packaging in our study obtained low dermal responses as expected, but surprisingly it obtained low evaluations in the questionnaire (instead of outstanding ones).
3. Electromyography (low-order emotion) and the items’ evaluation on the questionnaire (high-order emotion) were also inversely correlated (Table 4), while positive correlations were expected based on previous literature [18].That is, if eye movement acceleration is associated with the extent to which individuals increase attention to potentially interesting stimuli (literature, it is expected that packaging with quicker eye movements will also obtain higher evaluations on a questionnaire (better cognitive opinions). For the most chosen packaging in our study (blue packaging with elaborated claim), muscle activity was larger, as expected, because this was the preferred option, but curiously the opinions on the questionnaire for this packaging were not outstanding.

For the other five packages, most of the correlations between low-order emotions (unconscious responses) and high-order emotions (conscious responses) were not significant. Therefore, we conclude that low-order emotions and high-order emotions will not converge to explain product trials. H2 is accepted, as far as low-order emotions and high-order emotions did not converge to explain final product choices. 

Of even greater importance, the power of low-order emotions to anticipate product choices proved to be higher than the relevance of high-order emotions, because unconscious responses for the most chosen packaging followed the expected pattern, while opinions on the questionnaire proved to be not useful.

## 4. Discussion and Limitations

### 4.1. Discussion

Different packaging cues should be managed to stimulate consumption in the low-fat food industry. Four main theoretical conclusions have been obtained.

First, our results proved that pre-trial virtual stimulus can affect physical product choices. So, as in the work of [2]), we have demonstrated that virtual experience with a product enhanced consumer learning, so that it can be considered as an alternative consumer experience, suggesting that it resembles a product trial. In the words these authors, pre-trial of a virtual stimulus representing a product used in our first experiment was shown to increase the post-trial of this product in our second experiment.

Second, in regard to the stability of low-order emotions, our results demonstrated that physiological responses towards low-fat products do not change when passing from a virtual to a physical environment. Thus, pre-exposure towards virtual healthy products using screen presentations will anticipate real emotions towards real healthy products shown in a physical shop.

Third, depending on the environment (virtual or physical), the relevance of aesthetic cues (color) versus informative cues (message) could differ. In virtual environments, our results proved the stronger power of “informative cues” (messages on a label) over “aesthetic cues” (color) to promote low-fat products. The two most chosen virtual stimuli shared a common elaborated message (low-fat plus the image of a leaf). In contrast, in physical environments aesthetic cues (color) were more important than informative cues (messages) to promote a healthy product. In this context, the two most chosen real packages shared the same color: Blue. 

Finally, cognition (high-order emotions) was not helpful in explaining product choices. Even more, feelings (low-order emotions) and cognition (high-order emotions) did not act together. The most chosen packages received the worst evaluations on the questionnaire. That is, the most chosen packages did not have the highest values for valence, activation, or domination. So, although based on [11] both sources of information (unconscious and conscious) could be used in parallel to better understand consumer behavior, our results show the superiority of unconscious responses (low-order emotions) [25]. In this vein, our result support [6] proposal: “Individuals’ perceptions of their environment are frequently regulated by an unconscious mechanism that does not necessarily involve rational thinking”.

In sum, our results did not support the cognition-physiology school of thought, because perceptions about product packaging did not always have an impact on physiological responses. On the contrary, our results provided support for [26], stimulus–organism–response model (the M-R model), which dominates environmental psychology [27], as far as humans’ unconscious reactions dominate behavioral intentions. For this reason, although designers of healthy foods packaging can modify shoppers’ unconscious and conscious responses working on packaging cues, we recommend concentrating more effort on measuring shoppers’ unconscious reactions, as recommended also by [28].

### 4.2. Practical Implications

Based on our theoretical implications, the following managerial implications could be provided. 

First, our results show that in both scenes (virtual and physical), the most chosen option was the blue packaging with an elaborated claim (low-fat plus a leaf reinforcing the message). So, we recommend marketing managers of low-fat products the use of Internet and social networks to present new healthy products in advance using virtual simulations. This will improve potential users’ familiarity, because virtual and physical elections seem to be highly correlated.

Second, managers should not forget emphasizing the power of virtual environments to stimulate positive emotions among consumers. This is because when they enter physical shopping environments, what they have previously felt in the virtual world will affect what they finally feel towards real product presentations.

Third, we recommend emphasizing the message on the label when presenting a low-fat product virtually (elaborated claims instead of simple ones), and reinforce aesthetic cues (color) when showing the product in the real shop. 

Finally, we encourage product managers to use physiological data to anticipate consumers’ healthy behavior instead of merely relying on questionnaires before launching a new product.

### 4.3. Limitations

Finally, our study has some limitations that should be taken into account for future research. For example, additional cognitive measures could be included in the questionnaire to test further correlations between cognitive evaluations and low-order emotions. Additionally, other packaging design variables could be tested to obtain the perfect packaging in terms of sales. Targets other than young consumers (such as adolescents) could be analyzed. Also, more research is needed in the field of digital marketing to stimulate healthy consumption [29].

## Figures and Tables

**Table 1:** Convergence between virtual elections and real product elections.

Chosen Stimulus (Virtual)		Chosen Product (Physical)					
		Blue	Red	Black	BluePlume	RedPlume	BlackPlume
		22.08%	7.79%	2.60%	38.96%	19.48%	9.09%
Blue	3.6%	66.67%	0.00%	0.00%	33.33%	0.00%	0.00%
Red	7.2%	16.67%	33.33%	0.00%	16.67%	16.67%	16.67%
Black	3.6%	33.33%	0.00%	33.33%	33.33%	0.00%	0.00%
BluePlume	61.4%	20.83%	4.17%	2.08%	47.92%	18.75%	6.25%
RedPlume	10.8%	0.00%	12.50%	0.00%	12.50%	62.50%	12.50%
BlackPlume	13.3%	33.33%	11.11%	0.00%	33.33%	0.00%	22.22%

Association between virtual choices and physical choices: X^2^ = 40.493 (*p* < 0.02).

**Table 2:** Low-order emotions: Correlations between virtual stimulus and real products.

	HR		EDA		EMG	
	Max	Min	Max	Min	Basal Line	Average
Blue Stimulus and Blue Product	0.920 **	0.911 **	0.959 **	0.964 **	0.207 *	0.317 **
BluePlume Stimulus and BluePlume Product	0.928 **	0.914 **	0.965 **	0.969 **	0.084	0.109
Red Stimulus and Red Product	0.928 **	0.937 **	0.958 **	0.961 **	0.245 *	0.450 **
RedPlume Stimulus and RedPlume Product	0.918 **	0.557 **	0.967 **	0.967 **	0.336 **	0.339 **
Black Stimulus and Black Product	0.915 **	0.923 **	0.964 **	0.966 **	0.278 *	0.273 *
BlackPlume Stimulus and BlackPlume Product	0.937 **	0.944 **	0.956 **	0.965 **	0.189 *	0.292 **

** *p* < 0.01; * *p* < 0.05 EDA: Electro dermal Activity. HR: Heart rate. EMG: Electromyogram (*) Given that each stimulus/product was presented five times, an average of the maximums and minimums were calculated before estimating the correlations.

**Table 4:** Correlations between low-order emotions and high order emotions (cognitive items).

	**Blue Packaging**						**Blue + Plume Packaging**					
	**HR**		**EDA**		**EMG**		**HR**		**EDA**		**EMG**	
**ITEMS**	**Max**	**Min**	**Max**	**Min**	**Max**	**Min**	**Max**	**Min**	**Max**	**Min**	**Max**	**Min**
Valence1	−0.025	−0.080	−0.524 *	0.035	−0.117	−0.033	−0.135	−0.119	−0.135	−0.119	0.109	0.121
Activation 1	−0.111	−0.167	−0.221	0.085	0.283	0.173	0.181	0.292 *	0.181	0.292 *	−0.164	−0.150
Dominat1	0.052	0.032	−0.178	−0.123	−0.055	−0.038	−0.078	−0.085	−0.078	−0.085	−0.012	−0.008
Valence 2	0.423 *	0.356	−0.50 *	−0.422 *	0.068	0.123	0.040	0.087	0.040	0.087	−0.088	−0.084
Activation 2	0.050	0.015	−0.528 *	−0.052	0.118	0.001	0.439 *	0.461 *	0.439 *	0.461 *	−0.377 *	−0.368 *
Dominat 2	−0.036	−0.043	0.373	0.006	0.092	0.203	−0.304	−0.330	−0.304	−0.330	0.123	0.124
Valence 3	0.031	−0.118	−0.219	−0.053	0.060	0.070	0.529 **	0.586 **	0.529 **	0.586 **	−0.153	−0.138
Activation 3	−0.264	−0.328	−0.175	0.260	−0.127	−0.253	0.369 *	0.438 *	0.369 *	0.438 *	−0.541 **	−0.54 **
Dominat3	0.057	0.090	0.365	−0.071	−0.297	−0.133	−0.238	−0.296 *	−0.238	−0.296 *	0.039	0.038
	**Red Packaging**						**Red + Plume Packaging**					
	**HR**		**EDA**		**EMG**		**HR**		**EDA**		**EMG**	
**Cognitive Items**	**Max**	**Max**	**Max**	**Min**	**Max**	**Min**	**Max**	**Min**	**Max**	**Min**	**Max**	**Min**
Valence1	−0.572	−0.572	0.755 *	0.618	−0.455	−0.434	−0.524 *	−0.482 *	0.471 *	0.519 *	−0.037	−0.015
Activation 1	−0.460	−0.460	0.402	0.463	0.077	0.072	−0.221	−0.190	0.154	0.186	−0.146	−0.147
Dominat1	−0.229	−0.229	0.380	0.291	0.437	0.432	−0.178	−0.118	0.039	0.090	0.159	0.176
Valence 2	−0.532	−0.532	0.418	0.520	−0.547	−0.543	−0.50 *	−0.507 *	0.495 *	0.473 *	−0.097	−0.079
Activation 2	−0.402	−0.402	0.119	0.347	−0.295	−0.306	−0.528 *	−0.455 *	−0.510 *	0.485 *	0.442 *	0.184
Dominat 2	−0.75 *	−0.75 *	0.856 **	0.792 *	−0.168	−0.170	0.373	0.402	−0.464 *	−0.429 *	−0.231	−0.215
Valence 3	−0.505	−0.505	0.386	0.485	−0.411	−0.405	−0.219	−0.277	0.286	0.223	−0.168	−0.163
Activation 3	−0.358	−0.358	0.015	0.281	−0.366	−0.378	−0.175	−0.271	0.248	0.162	−0.048	−0.047
Dominat3	−0.028	−0.028	0.246	0.095	0.170	0.162	0.365	0.378	−0.414	−0.389	−0.509 *	−0.49 *
	**Black Packaging**						**Black + Plume Packaging**					
	**HR**		**EDA**		**EMG**		**HR**		**EDA**		**EMG**	
Valence1	n.a.	n.a.	n.a.	n.a.	n.a.	n.a.	0.134	0.074	−0.082	−0.146	−0.517	−0.510
Activation 1	n.a.	n.a.	n.a.	n.a.	n.a.	n.a.	0.574	0.717*	−0.67 *	−0.489	0.382	0.379
Dominat1	n.a.	n.a.	n.a.	n.a.	n.a.	n.a.	−0.029	0.177	−0.070	0.122	0.070	0.078
Valence 2	n.a.	n.a.	n.a.	n.a.	n.a.	n.a.	0.300	0.663 *	−0.582	−0.184	0.490	0.493
Activation 2	n.a.	n.a.	n.a.	n.a.	n.a.	n.a.	0.015	0.239	−0.184	0.050	0.909 **	0.915 **
Dominat 2	n.a.	n.a.	n.a.	n.a.	n.a.	n.a.	0.234	−0.249	0.119	−0.367	−0.207	−0.214
Valence 3	n.a.	n.a.	n.a.	n.a.	n.a.	n.a.	−0.338	0.176	−0.054	0.464	0.406	0.413
Activation 3	n.a.	n.a.	n.a.	n.a.	n.a.	n.a.	−0.515	−0.287	0.376	0.574	0.300	0.311
Dominat3	n.a.	n.a.	n.a.	n.a.	n.a.	n.a.	0.714 *	0.479	−0.566	−0.762 *	0.307	0.301

** *p* < 0.01; * *p* < 0.05. n.a. (not applicable) EDA: Electro dermal activity. HR: Heart rate. EMG: Electromyogram.
